# The effects of patient out‐of‐pocket costs on insulin use among people with type 1 and type 2 diabetes with Medicare Advantage insurance—2014–2018

**DOI:** 10.1111/1475-6773.14152

**Published:** 2023-03-29

**Authors:** Carrie McAdam‐Marx, Natalia Ruiz‐Negron, Jane M. Sullivan, Jamie M. Tucker

**Affiliations:** ^1^ Department of Pharmacy Practice & Science, Optum Labs, Visiting Fellow University of Nebraska Medical Center Omaha Nebraska USA; ^2^ Department of Pharmacotherapy University of Utah Pharmacotherapy Outcomes Research Center Salt Lake City Utah USA; ^3^ Optum Labs Eden Prairie Minnesota USA

**Keywords:** cost‐share, diabetes, insulin, Medicare Advantage, medication adherence

## Abstract

**Objective:**

To identify the association between insulin out‐of‐pocket costs (OOPC) and adherence to insulin in Medicare Advantage (MA) patients.

**Data Sources and Study Setting:**

The study is based on Optum Labs Data Warehouse, a longitudinal, real‐world data asset with de‐identified administrative claims and electronic health record data.

**Study Design:**

Using descriptive and multivariable logistic regression analyses, we identified the likelihood of patients with diabetes having ≥60 consecutive days between an expected insulin fill date and the actual fill date (refill lapse) by OOPC, categorized by $0, >$0–$20 (reference), >$20–$35, >$35–$50, and > $50 per 30‐day supply.

**Data Collection/Extraction Methods:**

The study included MA enrollees with type 1 or type 2 diabetes and prescription claims for insulin between 2014 and 2018.

**Principal Findings:**

Those with average insulin OOPC per 30‐day supply >$35 or $0 were more likely to have an insulin refill lapse versus OOPC of >$0 to $20, with odds ratios ranging 1.18 (95% CI 1.13–1.22) to 1.74 (95% CI 1.66–1.83) depending on OOPC group and diabetes type.

**Conclusions:**

Capping average insulin OOPC at $35 per 30‐day supply may help avoid cost‐related insulin non‐adherence in MA patients; efforts to address non‐cost barriers to medication adherence remain important.

**What is known on this topic**

- Insulin prices have increased dramatically over the past 15 years as have patient out‐of‐pocket costs (OOPC) for insulin.
- OOPC is a known barrier to diabetes medication adherence, including insulin.
- The Medicare Part D Innovations Senior Savings Model is a pilot program that limits insulin prescription OOPC to $35 per month for select insulins through participating enhanced Part D plans.

**What this study adds**

- Over 30% of Medicare Advantage enrollees taking insulin had a lapse of 60 or more consecutive days between the time an insulin prescription was due for a refill and the actual refill date.
- Those with average insulin OOPC per 30‐day supply of more than $35 were more likely to have an insulin refill lapse than those who paid out‐of‐pocket for insulin but at an amount of $20 or less.
- Capping insulin OOPC to no more than $35 per 30‐day supply may help to avoid cost‐related non‐adherence, but efforts to address other barriers to adherence continue to be important.

## 1 INTRODUCTION

In response to rising insulin prices and patient cost share,^1^, ^2^ states and payers have implemented policies limiting patients' share of insulin cost. By the end of 2021, 20 states had passed legislation capping insulin out‐of‐pocket cost (OOPC) in state regulated plans.^3^, ^4^, ^5^ Large commercial plans and pharmacy benefit managers (PBMs) also offer plans with insulin OOPC limits and/or support value‐based designs with low or reduced OOPC for diabetes medications including insulin.^6^, ^7^

The Medicare Part D Innovations Senior Savings Model also limits insulin OOPC. Started in 2021, this voluntary pilot program capped OOPC for select insulin products at $35 per 30‐day supply for patients in a participating enhanced Part D drug plan. At the time of this study, federal legislation to cap patients’ insulin OOPC for all Medicare beneficiaries, had been proposed but not passed.^8^, ^9^

Adherence to medications is complex and multifactorial,^10^ with OOPC a major factor. Thus, the goal in limiting patient OOPC is to specifically mitigate cost as a barrier to insulin adherence.^11^, ^12^ However, data on the impact of proposed or implemented insulin OOPC caps on insulin adherence in a Medicare population is limited. One historical cohort study conducted prior to the Senior Savings Model examined insulin adherence in enhanced Part D plan enrollees who entered the coverage gap. It found that insulin OOPC for enrollees in individual plans went from $51 in the initial coverage phase to $117 during the coverage gap with a corresponding decrease in adherence.^13^ Those in employer group waiver plans had steady insulin OOPC of approximately $32 and stable adherence as they transitioned from initial coverage to the coverage gap. The authors did not examine insulin adherence by OOPC and was limited to enrollees who entered the coverage gap.

The current study examined the association of insulin OOPC with lapses in insulin refills in Medicare Advantage (MA) enrollees regardless of whether they enter the coverage gap. This research aims to inform patient cost‐share policies designed to make insulin more affordable for Medicare patients.

## 2 METHODS

This retrospective cohort study included MA beneficiaries with type 1 (T1D) or type 2 (T2D) diabetes with analyses conducted by diabetes type using Optum Labs Data Warehouse data between January 1, 2013 and December 31, 2018 (before the Senior Savings Model). This dataset includes de‐identified medical and pharmacy claims, laboratory results, and enrollment records for commercial and MA enrollees. The database contains longitudinal health information on enrollees and patients in the United States.

Patient index date was January 1 from 2014 to 2018 following the first year in the study period in which the patient had two or more insulin fills dispensed at least 30 days apart, insulin in supply on November 1 prior to index date, and at least 1 insulin claim in the year following index date. Included patients had continuous enrollment in a MA plan with pharmacy coverage from a year before through a year after index date. Institutionalized patients receiving low‐income subsidy (LICS) covering 100% of insulin cost were excluded.

The study exposure was insulin OOPC during the 1‐year follow‐up period. In the absence of prescription drug benefit design data at the patient level, we estimated the average insulin OOPC during the follow‐up period based on the patient paid amount for insulin during the follow‐up year. When days’ supply dispensed for any insulin fill exceeded 30 days, OOPC was normalized to cost per 30‐day supply (e.g., OOPC for the fill divided by 30 days). Average OOPC per 30‐day supply for the year, regardless of coverage gap status, was categorized as $0, >$0–$20, >$20–$35, >$35–$50, or >$50.

The primary outcome was having at least one insulin refill lapse during the follow‐up year, defined as 60 or more consecutive days between the expected insulin refill date per days supply dispensed to the actual date of the refill. A medication supply gap is often used to determine treatment persistence, but almost all patients in this study with an insulin refill lapse had a subsequent claim for insulin during the follow‐up year. Therefore, a 60‐day refill lapse was more suggestive of non‐adherence than of non‐persistence.

This method recognizes that a late refill may not mean that a patient is without insulin the entire lapse period. Given insulin dose variability and product packaging (e.g., multidose vials; boxes of 5 pens), quantities dispensed likely cover more than the days supply reported on the claim. In addition, dose reductions would extend days supply. However, a claim for a 30‐day supply would need to have a 200% oversupply of insulin to cover a 60‐day refill lapse.

Secondary outcomes included the number of insulin claims and the daily average consumption (DACON) of insulin during the follow‐up period. DACON is the number of insulin units dispensed per day between the date of first insulin claim to the date of the last insulin claim during the follow‐up year. DACON is not based on reported days supply.

Baseline data included patient demographic information. Race and ethnicity were assigned by an external vendor based on names, surname prefixes, and suffixes with geographic criteria and then categorized into five compliance‐determined categories (non‐Hispanic White, non‐Hispanic Black, Hispanic, Asian, and unknown). Income and education data were estimated based on household and census block, respectively, while data on LICS status was as provided from Medicare Advantage claims data. Complications per the adapted Diabetes Complications Severity Index (aDCSI),^14^ and comorbidities per the Charlson Comorbidity Index (CCI)^15^ were captured as was baseline health care resource use and costs.

We used descriptive statistics to compare baseline characteristics and follow‐up measures pairwise to the >$0 to $20 OOPC group using chi‐square or Fishers exact test for categorical variables, and t‐test or rank sum for continuous variables. Multivariable logistic regression analyses were used to identify the odds of having a 60‐day insulin refill lapse during the 1‐year follow‐up period by average insulin OOPC per 30‐day supply versus >$0 to $20 OOPC. Models controlled for clinical, demographic, and socioeconomic factors that may influence the relationship between insulin OOPC and lapses in refills. We were not attempting to identify if social risk factors influence insulin use. Statistical analyses were performed at alpha of 0.05 using SAS version 9.4 and STATA 14 SE with no adjustment for multiple comparisons. The University of Nebraska Institutional Review Board determined that this study was not human subject research.

## 3 RESULTS

The study included 4023 individuals with T1D and 108,433 with T2D. Mean (sd) age of the T1D cohort was 65.6 (11.7) years, 45.4% were male, and mean baseline A1c was 8.06% (1.50) (*n* = 1495 had A1c data). Patients with T1D had an average of $555 (610) in total insulin OOPC during the 1‐year baseline period with an average baseline DACON of 62.8 (84.5) units/day. Of patients with T2D, mean age was 70.3 (9.0) years, 45.6% were male. Mean A1c was 8.25% (1.74) (*n* = 43,489 had A1c data), average insulin OOPC in the baseline year was $449 (588), and average baseline DACON was 57.3 (113.2) units/day.

Table 1 presents baseline characteristics by average insulin OOPC per 30‐day supply in the follow‐up year (hereafter referred to as OOPC). Over 50% of the T1D cohort had an average insulin OOPC of over $35, while 29% had OOPC of >$0–$20. A small proportion had $0 OOPC (6.7%). In the T2D cohort, just under 50% had an average insulin OOPC during the follow‐up year of over $35 whereas 38% had an OOPC of >$0 to $20; 4.6% had $0 OOPC. In both cohorts, the >$0–$20 OOPC group tended to be younger, with more comorbidity, higher insulin DACON, lower predicted income and education levels, and with a higher proportion receiving a LICS than other OOPC groups.

**TABLE 1:** Baseline characteristics—Medicare Advantage patients with diabetes by average insulin out of pocket cost per 30‐day supply.

	Average insulin out of pocket cost per 30‐day supply				
	$0	>$0–$20 (ref)	>$20–$35	>$35–$50	>$50
**Type 1 diabetes**					
**Number of patients (% of overall cohort)**	**269 (6.7)**	**1162 (28.9)**	**402 (10.0)**	**581 (14.4)**	**1609 (40.0)**
Age (mean, SD)	69.4 (9.2)**	58.3 (14)	70.6 (7.3)**	69.7 (8.8)**	67.6 (9.4)**
Sex (%)					
Male	48.0	44.1	39.8	43.6	47.9*
Female	52.0	55.9	60.2	56.5	52.1*
Estimated income (% of group)^a^					
<$40,000	21.9**	52.0	20.9**	18.9**	21.5**
$40,000–$74,999	33.5**	22.4	31.1**	20.8	30.5**
$75,000–$124,999	29.7**	12.4	27.9**	23.1**	29.8**
$125,000+	10.0**	4.4	14.2**	9.5**	13.2**
Unknown/Missing	4.8*	8.9	6.0	27.7**	5.0**
Low income subsidy (% of group)	7.1**	86.1	6.0**	6.4**	3.6**
Estimated education^a^					
High school diploma or less than 12th grade	>17.5**	55.9	29.9**	24.3**	30.1**
Less than Bachelor's Degree	62.8**	36.8	50.8**	39.6	55.9**
Bachelor's Degree or Higher	15.6**	5.9	14.9**	11.5**	11.9**
Unknown/Missing	<4.1	1.6	4.5**	24.6**	2.2
Predicted Race and Ethnicity (% of group)^b^					
Non‐Hispanic White	83.6**	63.1	75.9**	56.3*	79.6**
Non‐Hispanic Black	<4.1**	23.2	13.4**	12.1**	8.8**
Hispanic or Asian	>5.6*	10.6	4.2**	5.2**	6.7**
Unknown/Missing	6.7*	3.1	6.5*	26.5**	4.9*
Comorbidities (% of group with comorbidity)					
Hypertension	65.8	71.2	70.9	67.6	66.7*
Hyperlipidemia	52.0*	60.3	64.9	63.9	64.8*
ASCVD	37.9	37.3	38.6	38.7	34.9
Nephropathy	20.8	25.1	21.6	19.6*	20.7*
Retinopathy	35.7**	25.7	31.3*	27.0	27.5
Neuropathy	40.5	41.9	33.6*	32.7**	33.6**
aDCSI (mean, SD)	2.8 (2.2)	2.9 (2.3)	2.6 (2.3)	2.6 (2.4)*	2.6 (2.2)**
CCI (mean, SD)	1.9 (2)	1.8 (1.7)	1.8 (1.6)	1.7 (1.7)	1.6 (1.6)**
A1c %^c^ (mean, SD)	7.5 (1.4)*	8.5 (1.7)	7.8 (1.3)**	7.9 (1.4)**	7.9 (1.4)**
Baseline insulin DACON (mean, SD)	60.1 (44.7)*	69.4 (60.6)	66.2 (131.6)	59.3 (83.6)*	58.8 (89.4)**
Diabetes related costs baseline period (mean, SD)					
Patient out of pocket cost (mean, SD)					
Insulin	23 (83)**	104 (245)	583 (575)**	548 (345)**	965 (632)**
ED Visit	2 (11)**	18 (81)	4 (28)**	4 (22)**	5 (25)**
Inpatient stay	34 (252)**	252 (792)	50 (271)**	115 (655)**	102 (456)**
Office visit	30 (63)**	104 (144)	105 (106)	114 (247)	133 (271)**
Health plan paid cost (mean, SD)					
Insulin^d^	4496 (2929)**	5414 (4109)	4363 (3251)**	3824 (3089)**	2872 (2528)**
ED visit	51 (291)**	191 (893)	56 (345)**	56 (275)**	49 (283)**
Inpatient stay	451 (2252)**	3225 (19,432)	1779 (9096)*	1739 (8774)*	1338 (8348)*
Office visit	693 (1938)*	447 (599)	482 (695)	451 (1045)	475 (1101)
**Type 2 diabetes**					
**Number of patients (%of overall cohort)**	**4954 (4.6)**	**41,548 (38.3)**	**9099 (8.4)**	**15,247 (14.1)**	**37,585 (34.7)**
Age (mean, SD)	72.6 (8.8)**	67.2 (10.3)	73.0 (7.2)**	72.7 (7.6)**	71.9 (7.4)**
Sex (% of group)					
Male	47.0**	37.5	49.0**	48.2**	52.6**
Female	53.0**	62.5	51.0**	51.8**	47.4**
Estimated income^a^ (% of group)					
<$40,000	36.8**	58.3	29.3**	32.0**	33.0**
$40,000–$74,999	31.9**	22.8	32.0**	27.8**	33.0**
$75,000–$124,999	19.6**	7.6	23.8**	16.2**	21.5**
$125,000+	6.0**	2.1	8.3**	4.8**	7.5**
Unknown/Missing	5.8**	9.3	6.7**	19.2**	5.2**
Low income subsidy (% of group)	17.7**	88.6	8.3**	7.9**	3.9**
Estimated education^a^ (% of group)					
Less than 12th grade	0.4**	1.5	0.3**	0.3**	0.4**
High school diploma	31.8**	62.2	35.0**	40.2**	40.6**
Less than bachelor's degree	58.8**	32.6	52.0**	37.7**	50.0**
Bachelor's degree or higher	8.3**	2.9	9.0**	5.9**	7.0**
Unknown/Missing	0.8	0.8	3.7**	15.9**	1.9**
Predicted race and ethnicity (% of group)^b^					
Non‐Hispanic White	68.6**	44.3	66.6**	52.1**	69.3**
Non‐Hispanic Black	9.9**	33.2	18.1**	21.5**	14.6**
Hispanic or Asian	18.3**	20.5	9.4**	9.0**	12.0**
Unknown/Missing	3.3**	2.0	5.9**	17.4**	4.0**
Comorbidities (% of group with comorbidity)					
Hypertension	67.3**	87.4	85.1**	86.3**	84.4**
Hyperlipidemia	50.3**	68.9	70.7**	70.5**	70.9**
ASCVD	42.6**	50.1	51.1	49.2	48.2**
Nephropathy	29.9**	32.7	35.1**	32.7	30.6**
Retinopathy	12.1**	15.3	15.5	14.4*	14.4**
Neuropathy	26.8**	38.5	31.8**	29.8**	29.7**
aDCSI (mean, SD)	2.4 (2.3)**	2.9 (2.2)	2.9 (2.2)	2.7 (2.2)**	2.7 (2.2)**
CCI (mean, SD)	2.2 (2.2)**	2.4 (2)	2.4 (2.1)	2.2 (2.0)**	2.1 (1.9)**
A1c %^e^ (mean, SD)	8.0 (1.9)**	8.4 (1.9)	7.9 (1.5)**	8.1 (1.6)**	8.2 (1.7)**
Baseline insulin DACON (mean, SD)	57.8 (103.8)**	64.7 (116.9)	59.8 (176.6)*	47.9 (104.4)**	52.4 (91.4)**
Diabetes‐related costs baseline period (mean, SD)					
Patient out of pocket cost (mean, SD)					
Insulin	17 (108)**	84 (225)	532 (621)**	505 (413)**	868 (652)**
Non‐insulin diabetes medications	7 (56)**	34 (120)	204 (374)**	151 (318)**	185 (387)**
ED visit	2 (18)**	7 (34)	2 (14)**	3 (18)**	3 (18)**
Inpatient stay	41 (281)**	124 (553)	46 (269)**	71 (385)**	102 (483)**
Office visit	22 (61)**	88 (228)	79 (160)**	79 (153)**	88 (191)
Health plan paid cost (mean, SD)					
Insulin^d^	3769 (3866)**	4764 (4691)	3901 (4436)**	2857 (3469)**	2291 (2696)**
ED visit	20 (158)**	67 (376)	28 (247)**	34 (219)**	30 (251)**
Inpatient stay	938 (9044)**	1647 (9574)	1486 (9644)	1335 (8165)**	1649 (12,209)
Office visit	336 (892)**	408 (1048)	451 (1228)*	399 (1083)	400 (882)

* Pairwise *p*‐value <0.05 versus >$0–$20 out‐of‐pocket cost group.

** Pairwise *p*‐value ≤0.001 versus >$0–$20 out‐of‐pocket cost group.

^a^ Income estimated at household level and education estimated from census block.

^b^ Ethnicity is assigned by an external vendor who uses a rule‐based system that combines analysis of first names, middle names, surnames, and surname prefixes and suffixes with geographic criteria. Optum Labs then assigns these ethnicity values into one of five compliance‐determined race code values: W (Non‐Hispanic White), B (Non‐Hispanic Black), H (Hispanic), A (Asian), and U (Unknown).

^c^ In 1495 patients with A1c value at baseline.

^d^ Includes costs for insulin not paid by patient, for example, health plan paid, low‐income subsidy payments, and other sources.

^e^ In 43,489 patients with A1c value at baseline.

Abbreviations: aDCSI, adjusted Diabetes Complication Severity Index; ASCVD, atherosclerotic cardiovascular disease; CCI, Charlson Comorbidity Index; ED, emergency department; mPDC, modified proportion of days covered.

### 3.1 Insulin refill lapse

In the T1D cohort, 37.1% had an insulin refill lapse during the follow‐up year (Table 2). The proportion with a refill lapse was significantly higher for those with an average insulin OOPC >$35 to $50 (38.6%) and over $50 (41.5%) versus >$0 to $20 (32.3%; *p* < 0.01 for both). In multivariable analyses, individuals with OOPC >$35 to $50 (odds ratio [OR] 1.50; 95% CI 1.15,1.95) or over $50 (OR 1.40, 95% CI 1.12, 1.75) were more likely to experience an insulin refill lapse vs. those with OOPC of >$0 to $20, as were those in the $0 OOPC (OR 1.56; 95% CI 1.10, 2.21); this was not observed for those in >$20 to $35 OOPC group (OR 1.21; CI 0.90, 1.62). (Table 3, Table S1).

**TABLE 2:** Insulin and antihyperglycemic medication utilization measures by average insulin out of pocket cost per 30‐day supply.

	$0	>$0–$20 (Ref)	>$20–$35	>$35–$50	>$50
**Type 1 diabetes**					
Number of patients	269	1162	402	581	1609
With a 60‐day insulin refill lapse (%)	95 (35.3)	375 (32.3)	130 (32.3)	224 (38.6)*	668 (41.5)**
With a 90‐day insulin refill lapse sensitivity analyses (%)	49 (18.2)	230 (19.8)	76 (18.9)	138 (23.8)	407 (25.3)**
Number of insulin claims (mean, SD)	8.0 (6.1)	8.5 (5.1)	7.1 (4.9)**	7.6 (5.5)*	7.3 (4.9)**
Insulin DACON (mean, SD)	57.2 (34.5)**	76.4 (160.8)	58.9 (48.0)**	66.4 (184.8)	63.9 (133.5)*
25% decrease in DACON from baseline (%)	14.1	18.3	19.7	16.9	15.1*
**Type 2 diabetes**					
Number of patients	4954	41,548	9099	15,247	37,585
With a 60‐day insulin refill lapse (%)	2032 (41.0)**	15,487 (37.3)	3275 (36.0)*	7554 (49.5)**	16,000 (42.6)**
With a 90‐day insulin refill lapse sensitivity analyses (%)	1361 (27.5)**	9917 (23.9)	2103 (23.1)	5302 (34.8)**	10,279 (27.4)**
Number of insulin claims (mean, SD)	8.9 (7.1)**	8.0 (5.5)	7.3 (5.5)**	6.6 (5.0)**	7.4 (4.9)**
Insulin DACON (mean, SD)	59.0 (104.7)**	68.4 (146.3)	63.3 (174.0)*	48.7 (136.8)**	54.9 (122.8)**
25% decrease in DACON from baseline (%)	18.7*	20.2	19.0*	22.9**	17.8**

* *p*‐value <0.05 vs. >$0–$20 average out‐of‐pocket cost category.

** *p*‐value <0.001 vs. >$0–$20 average out‐of‐pocket cost category.

Abbreviations: DACON, daily average consumption in insulin units; SD, standard deviation.

**TABLE 3:** Odds of 60‐day lapse in insulin refills by insulin out‐of‐pocket cost category, relative to >$0–$20 out‐of‐pocket cost^a^

	Odds ratio (95% confidence interval)	
	60‐day insulin refill lapse	
	Type 1 diabetes	Type 2 diabetes
$0	1.56 (1.10, 2.21)	1.33 (1.24, 1.43)
>$0–$20	Ref	Ref
>$20–$35	1.21 (0.90, 1.62)	1.11 (1.05, 1.17)
>$35–$50	1.50 (1.15, 1.95)	1.74 (1.66, 1.83)
>$50	1.40 (1.12, 1.75)	1.18 (1.13, 1.22)

Type 1 Diabetes *N* = 4023; Type 2 Diabetes *N* = 108,433.

^a^ Models controlled for baseline age, gender, predicted race and ethnicity, region, index date year, estimated income and education, comorbidities, utilization, and cost (insulin, outpatient care, office visits, hospitalization, and emergency department), non‐insulin antihyperglycemic medication cost and adherence, and insulin daily average consumption (DACON).

In the T2D cohort, 40.9% overall had an insulin refill lapse during the follow‐up year (Table 2). The proportion with a refill lapse was significantly higher in those with OOPC >$35 to $50 (49.5%) and over $50 (42.6%), as well as those with $0 OOPC (41.0%) versus >$0 to $20 OOPC (37.3%) (*p* < 0.001 for all). However, the proportion with a refill lapse was less in the >$20 to $35 OOPC group (36.0%; *p* = 0.022). In multivariable analyses, all OOPC groups had a higher likelihood having a refill lapse vs. the >$0 to $20 group with OR ranging from 1.11 (95% CI 1.05, 1.17) in the >$20 to $35 group to 1.74 (95% CI 1.66, 1.83) in the >$35 to $50 group (Table 3, Table S2).

Table 2 provides additional detail on insulin use in the follow‐up year. In the T1D cohort, the number of insulin claims dispensed was greater in the >$0 to $20 group than groups with OOPC more than $20 (*p* < 0.05). Follow‐up DACON was higher in the >$0 to $20 group than other OOPC groups (*p* < 0.05) except the >$35 to $50 OOPC group. In the T2D cohort, the average number of claims were higher in the >$0 to $20 OOPC group compared with OOPC groups of over $20 (*p* < 0.05), but lower than in the $0 OOPC group (*p* < 0.001). DACON was higher in the >$0 to $20 OOPC group.

## 4 DISCUSSION

Insulin is a mainstay of diabetes care, contributing to a treatment goal of near‐normal blood glucose levels to prevent diabetes‐related complications. Insulin is generally essential therapy for those with T1D, and it is recommended for those with T2D whose blood glucose is uncontrolled while on other diabetes therapies.^16^ Thus, removing barriers to insulin use, including cost, is key to avoiding poor diabetes outcomes.

This nationwide study of MA beneficiaries with diabetes between 2013 and 2018 examined if OOPC is associated with reduced insulin adherence. It found that those with T1D or T2D and average insulin OOPC over $35 per 30‐day supply had a higher likelihood of having an insulin refill lapse of 60 or more consecutive days than those with OOPC of >$0 to $20. In T2D, those with an OOPC of >$20 to $35 were also more likely to have a refill lapse.

An unexpected finding was that having no OOPC for insulin in the follow‐up year was associated with a higher likelihood of having an insulin refill lapse versus an OOPC of up to $20. Examination of dispensing details by OOPC group did not suggest “stockpiling” by those with a low OOPC, which could impact refill frequency (data not shown). Non‐adherence in the $0 OOPC group may have been driven by factors not related to cost and assessed in this study, such as perceived benefits/risks of insulin therapy, transportation issues, or low health literacy. Although additional research is warranted to shed light on this finding, it is a reminder of the importance of addressing all barriers to medication adherence.

Overall, the study findings suggest that a $35 cap on insulin OOPCmay help to reduce the risk non‐adherence to insulin for many patients, and that a lower cap may be beneficial in those with T2D and who may be more price sensitive to insulin given that they may have non‐insulin treatment options. In its inaugural year (2021), it was estimated that the Senior Savings Model would reduce insulin OOPC for beneficiaries of participating plans by 66%.^17^ However, an analyses of Part D premium trends for standalone prescription drug plans for 2022 suggest that a substantial portion of patient savings could be offset by higher premiums.^18^ This is an important policy consideration that will require solutions among multiple stakeholders, as simply shifting costs from insulin cost‐share to premiums may not protect patients from non‐adherence due to cost.

### 4.1 Limitations

This study has limitations that warrant consideration. As noted previously, quantifying insulin adherence based on administrative claims data is prone to measurement bias due to inaccuracies in reported days supply. Our adherence measurement approach, a refill lapse of 60 or more consecutive days, was based on days supply but is a conservative estimate. In a sensitivity analysis, we examined refill lapse based on a 90‐day threshold (Table 2). Although fewer patients had a refill lapse, the association between OOPC and refill lapse generally remained. We also did not assess refill lapses by insulin type, vial versus syringe delivery, or by insulin pump use which may affect adherence.

In addition, the data used for this study did not include specific details about MA enrollee pharmacy benefits. Thus, we estimated insulin OOPC from actual patient paid amounts during the follow‐up year. This approach may mask changes in adherence as coverage and actual OOPC changes. In addition, administrative claims data do not capture information on insulin purchased with cash or not claimed through the patients' MA plan. Thus, we may overestimate insulin non‐adherence for patients purchasing insulin outside their MA benefit. Further, this study did not consider other health care OOPC or premiums, which may differ by OOPC group and could affect insulin adherence. Finally, several estimated demographic variables were included largely to describe the cohort. This includes income and education level, estimated using census data, as well as race and ethnicity data, which were imputed using a validated, proprietary algorithm. Misclassification of these variables is, therefore, plausible, and, interpretation of findings related to these variable must be approached with caution. Research specifically designed to investigate how social risk factors influence cost‐related non‐adherence to insulin is warranted.

Despite limitations, this study contributes to the literature by including those who enter the coverage gap and non‐institutionalized LICS patients, and in assessing adherence per categories of OOPC to determine how ranges of average OOPC affect adherence. In the context of other findings, this study can be used to inform coverage policies aimed at establishing an optimal insulin OOPC limit in a MA population. It further supports that protecting MA patients from high insulin OOPC over the course of a year may help to avoid cost‐related non‐adherence to insulin while also serving as a reminder that factors other than cost can affect adherence. Studies that directly examine the impact of the Senior Savings Model on insulin adherence and long‐term outcomes are needed.

In conclusion, this large, nationwide study of MA patients with diabetes found that insulin refill lapses were more likely to occur for those with T1D who paid more than $35 per 30‐day supply of insulin on average during the follow‐up year and more than $20 for those with T2D. This study provides evidence in support of policies to limit insulin OOPC and help avoid cost‐related non‐adherence. However, OOPC is not the only driver of medication adherence, and efforts to address all adherence barriers continue to be important.

## Supporting information


**Table S1.** Odds of having a 60‐day gap in insulin supply by average insulin out‐of‐pocket cost per 30‐day supply, multivariable logistic regression, Medicare Advantage, type 1 diabetes
**Table S2.** Odds of having a 60‐day gap in insulin supply by average insulin out‐of‐pocket cost per 30‐day supply, multivariable logistic regression, Medicare Advantage, type 2 diabetes
