# Supplementary material for: The effects of patient out‐of‐pocket costs on insulin use among people with type 1 and type 2 diabetes with Medicare Advantage insurance—2014–2018
Source: Health Serv Res. 2023 Mar 29;59(1):e14152. doi: 10.1111/1475-6773.14152 (PMC10771896; doi:10.1111/1475-6773.14152)
Supplement: Supplementary file 1 — Table S1. Odds of having a 60‐day gap in insulin supply by average insulin out‐of‐pocket cost per 30‐day supply, multivariable logistic regression, Medicare Advantage, type 1 diabetes Table S2. Odds of having a 60‐day gap in insulin supply by average insulin out‐of‐pocket cost per 30‐day supply, multivariable logistic regression, Medicare Advantage, type 2 diabetes [file HESR-59-e14152-s001.docx]

**Supplemental Materials**

Acknowledgement: This study was funded by the Robert Wood Johnson Foundation, Health Data for Action Research Program. The study sponsor played no role in the design or conduct of this study. The views expressed here do not necessarily reflect the views of the Foundation.

Appendix Table A1: Odds of Having a 60-Day Gap in Insulin Supply by Average Insulin Out-of-Pocket Cost per 30-Day Supply, Multivariable Logistic Regression, Medicare Advantage, Type 1 Diabetes

Appendix Table A2: Odds of Having a 60-Day Gap in Insulin Supply by Average Insulin Out-of-Pocket Cost per 30-Day Supply, Multivariable Logistic Regression, Medicare Advantage, Type 2 Diabetes

**Appendix Table A1: Odds of Having a 60-Day Gap in Insulin Supply by Average Insulin Out-of-Pocket Cost per 30-Day Supply, Multivariable Logistic Regression, Medicare Advantage, Type 1 Diabetes**

| **60-Day Gap in Insulin** | **Odds Ratio** | **Std Err** | **z** | **P-Value** | **95% Conf. Interval** | |
| --- | --- | --- | --- | --- | --- | --- |
| Insulin Out-of-Pocket Cost (ref >$0-$20) | |  |  |  |  |  |
| $0 | 1.562 | 0.277 | 2.510 | 0.012 | 1.103 | 2.210 |
| >$20-$35 | 1.207 | 0.181 | 1.260 | 0.209 | 0.900 | 1.618 |
| >$35-$50 | 1.498 | 0.201 | 3.020 | 0.003 | 1.152 | 1.948 |
| >$50 | 1.403 | 0.159 | 2.980 | 0.003 | 1.123 | 1.752 |
| Age (years) | 0.987 | 0.004 | -3.680 | <0.001 | 0.980 | 0.994 |
| Sex (ref Male) | 0.901 | 0.063 | -1.470 | 0.140 | 0.785 | 1.035 |
| Predicted Race and Ethnicity^a^ (ref. non-Hispanic White) | | |  |  |  |  |
| Non-Hispanic Black | 1.054 | 0.113 | 0.490 | 0.622 | 0.855 | 1.300 |
| Hispanic | 1.309 | 0.190 | 1.850 | 0.064 | 0.985 | 1.741 |
| Asian | 1.196 | 0.344 | 0.620 | 0.533 | 0.681 | 2.101 |
| Unknown/Missing | 0.752 | 0.175 | -1.230 | 0.220 | 0.477 | 1.185 |
| Region (ref Northeast) |  |  |  |  |  |  |
| Midwest | 0.924 | 0.112 | -0.650 | 0.515 | 0.728 | 1.172 |
| South | 1.118 | 0.121 | 1.030 | 0.303 | 0.904 | 1.382 |
| West | 1.041 | 0.154 | 0.280 | 0.783 | 0.780 | 1.391 |
| Year of Index Date (ref 2014) |  |  |  |  |  |  |
| 2015 | 0.975 | 0.136 | -0.180 | 0.858 | 0.742 | 1.282 |
| 2016 | 0.898 | 0.126 | -0.770 | 0.442 | 0.682 | 1.182 |
| 2017 | 0.847 | 0.107 | -1.320 | 0.187 | 0.662 | 1.084 |
| 2018 | 0.909 | 0.117 | -0.740 | 0.460 | 0.707 | 1.170 |
| Estimated Income^b^ (ref <$40,000) | |  |  |  |  |  |
| $40,000-$74,999 | 0.939 | 0.088 | -0.660 | 0.506 | 0.781 | 1.130 |
| $75,000-$124,999 | 1.003 | 0.105 | 0.020 | 0.981 | 0.816 | 1.232 |
| $125,000-$199,999 | 0.834 | 0.132 | -1.150 | 0.252 | 0.611 | 1.138 |
| $200,000+ | 0.843 | 0.195 | -0.740 | 0.460 | 0.535 | 1.327 |
| Missing/unknown | 1.022 | 0.186 | 0.120 | 0.907 | 0.715 | 1.460 |
| Estimated Education^b^ (ref High School Diploma) | |  |  |  |  |  |
| Less than 12th grade | 1.775 | 1.117 | 0.910 | 0.362 | 0.517 | 6.096 |
| Less than Bachelor’s Degree | 1.027 | 0.086 | 0.320 | 0.748 | 0.872 | 1.210 |
| Bachelor’s Degree or Higher | 1.091 | 0.155 | 0.620 | 0.538 | 0.827 | 1.441 |
| Unknown/Missing | 0.925 | 0.306 | -0.230 | 0.815 | 0.484 | 1.771 |
| Low Income Subsidy | 1.100 | 0.082 | 1.280 | 0.202 | 0.950 | 1.273 |
| Comorbidities at Baseline |  |  |  |  |  |  |
| aDCSI | 1.028 | 0.027 | 1.040 | 0.297 | 0.976 | 1.082 |
| CCI | 0.982 | 0.028 | -0.640 | 0.520 | 0.928 | 1.039 |
| Retinopathy | 1.039 | 0.089 | 0.440 | 0.659 | 0.878 | 1.229 |
| Neuropathy | 1.099 | 0.091 | 1.140 | 0.256 | 0.934 | 1.293 |
| Nephropathy | 0.760 | 0.083 | -2.520 | 0.012 | 0.614 | 0.941 |
| Myocardial Infarction | 1.500 | 0.427 | 1.420 | 0.155 | 0.858 | 2.621 |
| Chronic Kidney Disease | 1.251 | 0.110 | 2.540 | 0.011 | 1.053 | 1.487 |
| Depression | 1.098 | 0.102 | 1.000 | 0.316 | 0.915 | 1.318 |
| Heart Failure | 1.103 | 0.161 | 0.670 | 0.504 | 0.828 | 1.469 |
| Hyperlipidemia | 0.882 | 0.066 | -1.670 | 0.095 | 0.760 | 1.022 |
| Hypertension | 1.098 | 0.089 | 1.150 | 0.249 | 0.937 | 1.287 |
| Ischemic Heart Disease | 1.015 | 0.093 | 0.160 | 0.870 | 0.848 | 1.215 |
| Stroke | 1.015 | 0.210 | 0.070 | 0.941 | 0.677 | 1.524 |
| Utilization (by event) |  |  |  |  |  |  |
| ED Visit | 1.003 | 0.051 | 0.050 | 0.961 | 0.999 | 1.002 |
| Hospitalizations | 0.916 | 0.081 | -1.000 | 0.320 | 1.000 | 1.000 |
| Office Visits | 0.997 | 0.004 | -0.830 | 0.405 | 1.000 | 1.000 |
| Outpatient Visits | 1.001 | 0.002 | 0.470 | 0.638 | 1.000 | 1.000 |
| Patient Out-of-Pocket Costs |  |  |  |  |  |  |
| Non-Insulin AHG Drugs | 1.000 | 0.000 | 0.680 | 0.494 | 1.000 | 1.001 |
| ED Visits | 1.001 | 0.001 | 0.900 | 0.367 | 0.999 | 1.002 |
| Hospitalizations | 1.000 | 0.000 | 0.900 | 0.366 | 1.000 | 1.000 |
| Office Visits | 1.000 | 0.000 | 0.470 | 0.637 | 1.000 | 1.000 |
| Outpatient Visits | 1.000 | 0.000 | 0.870 | 0.382 | 1.000 | 1.000 |
| Health Plan Paid Costs |  |  |  |  |  |  |
| ED Visits | 1.000 | 0.000 | -1.310 | 0.189 | 1.000 | 1.000 |
| Hospitalizations | 1.000 | 0.000 | -1.070 | 0.283 | 1.000 | 1.000 |
| Office Visits | 1.000 | 0.000 | 0.380 | 0.704 | 1.000 | 1.000 |
| Outpatient Visits | 1.000 | 0.000 | 0.060 | 0.951 | 1.000 | 1.000 |
| Insulin* | 1.000 | 0.000 | -9.710 | <0.001 | 1.000 | 1.000 |
| Baseline Insulin DACON | 1.000 | 0.000 | 0.210 | 0.830 | 0.999 | 1.001 |
| Constant | 1.953 | 0.598 | 2.190 | 0.029 | 1.072 | 3.558 |

^*^Includes costs for medications not paid by patient e.g., health plan paid, low-income subsidy payments, and other sources

aDCSI – Adjusted Diabetes Complication Severity Index, CCI – Charlson Comorbidity Index, ED – Emergency Department, DACON – Daily Average Consumption in insulin units

a. Income and education estimated from census division.

b. Ethnicity is assigned by an external vendor who uses a rule-based system that combines analysis of first names, middle names, surnames, and surname prefixes and suffixes with geographic criteria. Optum Labs then assigns these ethnicity values into one of five compliance-determined race code values: W (Non-Hispanic White), B (Non-Hispanic Black), H (Hispanic), A (Asian), and U (Unknown).”

Number of obs: 4,023

p <.001

Pseudo R2: 0.049

**Appendix Table A2: Odds of Having a 60-Day Gap in Insulin Supply by Average Insulin Out-of-Pocket Cost per 30-Day Supply, Multivariable Logistic Regression, Medicare Advantage, Type 2 Diabetes**

| **60-Day Gap in Insulin** | **Odds Ratio** | **Std Err** | **z** | **P-Value** | **95% Conf. Interval** | |
| --- | --- | --- | --- | --- | --- | --- |
| Insulin Out-of-Pocket Cost (ref >$0-$20) | |  |  |  |  |  |
| $0 | 1.330 | 0.047 | 8.080 | <0.001 | 1.241 | 1.426 |
| >$20-$35 | 1.112 | 0.031 | 3.770 | <0.001 | 1.052 | 1.175 |
| >$35-$50 | 1.745 | 0.042 | 23.060 | <0.001 | 1.664 | 1.829 |
| >$50 | 1.176 | 0.024 | 7.930 | <0.001 | 1.130 | 1.225 |
| Age (years) | 0.982 | 0.001 | -22.220 | <0.001 | 0.981 | 0.984 |
| Sex (ref Male) | 1.021 | 0.014 | 1.580 | 0.115 | 0.995 | 1.049 |
| Predicted Race and Ethnicity^a^ (ref. non-Hispanic White) | |  |  |  |  |  |
| Non-Hispanic Black | 1.199 | 0.021 | 10.510 | <0.001 | 1.159 | 1.240 |
| Hispanic | 1.240 | 0.026 | 10.280 | <0.001 | 1.190 | 1.291 |
| Asian | 1.255 | 0.057 | 5.030 | <0.001 | 1.148 | 1.371 |
| Unknown/Missing | 0.975 | 0.047 | -0.530 | 0.598 | 0.888 | 1.071 |
| Region (ref Northeast) |  |  |  |  |  |  |
| Midwest | 0.955 | 0.022 | -1.960 | 0.050 | 0.913 | 1.000 |
| South | 1.064 | 0.022 | 2.990 | 0.003 | 1.022 | 1.108 |
| West | 1.119 | 0.034 | 3.700 | <0.001 | 1.054 | 1.187 |
| Year of Index Date (ref 2014) |  |  |  |  |  |  |
| 2015 | 0.929 | 0.025 | -2.790 | 0.005 | 0.882 | 0.978 |
| 2016 | 0.997 | 0.026 | -0.120 | 0.902 | 0.947 | 1.049 |
| 2017 | 0.934 | 0.022 | -2.880 | 0.004 | 0.892 | 0.979 |
| 2018 | 1.010 | 0.025 | 0.410 | 0.680 | 0.963 | 1.060 |
| Estimated Income^b^ (ref <$40,000) | |  |  |  |  |  |
| $40,000-$74,999 | 0.936 | 0.015 | -4.080 | <0.001 | 0.907 | 0.966 |
| $75,000-$124,999 | 0.930 | 0.020 | -3.440 | 0.001 | 0.893 | 0.969 |
| $125,000-$199,999 | 0.950 | 0.035 | -1.410 | 0.159 | 0.885 | 1.020 |
| $200,000+ | 1.171 | 0.075 | 2.470 | 0.013 | 1.033 | 1.327 |
| Unknown/Missing | 0.991 | 0.029 | -0.290 | 0.770 | 0.935 | 1.051 |
| Estimated Education^b^ (ref High School Diploma) | |  |  |  |  |  |
| Less than 12^th^ grade | 1.109 | 0.080 | 1.440 | 0.150 | 0.963 | 1.278 |
| Less than Bachelor’s Degree | 0.987 | 0.015 | -0.880 | 0.378 | 0.959 | 1.016 |
| Bachelor’s Degree or Higher | 0.953 | 0.031 | -1.520 | 0.129 | 0.895 | 1.014 |
| Unknown/Missing | 0.695 | 0.045 | -5.630 | <0.001 | 0.612 | 0.789 |
| Low Income Subsidy | 1.008 | 0.013 | 0.640 | 0.522 | 0.983 | 1.034 |
| Comorbidities at Baseline |  |  |  |  |  |  |
| aDCSI | 1.023 | 0.005 | 4.580 | <0.001 | 1.013 | 1.034 |
| CCI | 1.002 | 0.005 | 0.440 | 0.656 | 0.993 | 1.011 |
| Retinopathy | 0.934 | 0.019 | -3.420 | 0.001 | 0.898 | 0.971 |
| Neuropathy | 1.062 | 0.017 | 3.870 | <0.001 | 1.030 | 1.095 |
| Nephropathy | 0.922 | 0.018 | -4.080 | <0.001 | 0.887 | 0.959 |
| Myocardial Infarction | 0.922 | 0.043 | -1.760 | 0.078 | 0.842 | 1.009 |
| Chronic Kidney Disease | 1.043 | 0.019 | 2.330 | 0.020 | 1.007 | 1.081 |
| Depression | 1.084 | 0.019 | 4.610 | <0.001 | 1.048 | 1.122 |
| Heart Failure | 1.064 | 0.023 | 2.930 | 0.003 | 1.021 | 1.109 |
| Hyperlipidemia | 0.958 | 0.014 | -2.880 | 0.004 | 0.930 | 0.986 |
| Hypertension | 0.939 | 0.019 | -3.210 | 0.001 | 0.903 | 0.976 |
| Ischemic Heart Disease | 1.024 | 0.017 | 1.490 | 0.136 | 0.992 | 1.057 |
| Stroke | 1.003 | 0.031 | 0.100 | 0.924 | 0.945 | 1.065 |
| Utilization (by event) |  |  |  |  |  |  |
| ED Visit | 1.023 | 0.009 | 2.540 | 0.011 | 1.000 | 1.000 |
| Hospitalizations | 1.048 | 0.015 | 3.160 | 0.002 | 1.000 | 1.000 |
| Office Visits | 0.998 | 0.001 | -3.010 | 0.003 | 1.000 | 1.000 |
| Outpatient Visits | 1.000 | 0.000 | -0.590 | 0.555 | 1.000 | 1.000 |
| Patient Out-of-Pocket Costs |  |  |  |  |  |  |
| Non-Insulin AHG Drugs | 1.000 | 0.000 | -8.900 | <0.001 | 1.000 | 1.000 |
| ED Visits | 1.000 | 0.000 | 0.490 | 0.626 | 1.000 | 1.000 |
| Hospitalizations | 1.000 | 0.000 | 1.600 | 0.111 | 1.000 | 1.000 |
| Office Visits | 1.000 | 0.000 | 2.380 | 0.017 | 1.000 | 1.000 |
| Outpatient Visits | 1.000 | 0.000 | 1.210 | 0.225 | 1.000 | 1.000 |
| Health Plan Paid Costs |  |  |  |  |  |  |
| ED Visits | 1.000 | 0.000 | 1.480 | 0.138 | 1.000 | 1.000 |
| Hospitalizations | 1.000 | 0.000 | 0.840 | 0.400 | 1.000 | 1.000 |
| Office Visits | 1.000 | 0.000 | 1.470 | 0.142 | 1.000 | 1.000 |
| Outpatient Visits | 1.000 | 0.000 | 3.320 | 0.001 | 1.000 | 1.000 |
| Insulin* | 1.000 | 0.000 | -58.020 | <0.001 | 1.000 | 1.000 |
| Non-Insulin Diabetes Drugs* | 1.000 | 0.000 | -2.220 | 0.027 | 1.000 | 1.000 |
| Baseline Adherence to Non-Insulin Diabetes Drug | 0.827 | 0.015 | -10.220 | <0.001 | 0.797 | 0.858 |
| Baseline Insulin DACON | 1.001 | 0.000 | 6.470 | <0.001 | 1.000 | 1.001 |
| Constant | 3.196 | 0.224 | 16.550 | <0.001 | 2.785 | 3.667 |

^*^Includes costs for medications not paid by patient e.g., health plan paid, low-income subsidy payments, and other sources

aDCSI – Adjusted Diabetes Complication Severity Index, CCI – Charlson Comorbidity Index, ED – Emergency Department, DACON – Daily Average Consumption in insulin units

a. Income and education estimated from census division.

b. Ethnicity is assigned by an external vendor who uses a rule-based system that combines analysis of first names, middle names, surnames, and surname prefixes and suffixes with geographic criteria. Optum Labs then assigns these ethnicity values into one of five compliance-determined race code values: W (Non-Hispanic White), B (Non-Hispanic Black), H (Hispanic), A (Asian), and U (Unknown).”

Number of obs: 108,433

p <0.001

Pseudo R2: 0.054
